# Recessive Resistance to Plant Viruses: Potential Resistance Genes Beyond Translation Initiation Factors

**DOI:** 10.3389/fmicb.2016.01695

**Published:** 2016-10-26

**Authors:** Masayoshi Hashimoto, Yutaro Neriya, Yasuyuki Yamaji, Shigetou Namba

**Affiliations:** Laboratory of Plant Pathology, Department of Agricultural and Environmental Biology, Graduate School of Agricultural and Life Sciences, The University of TokyoTokyo, Japan

**Keywords:** plant virus disease control, host resistance, recessive resistance, translation initiation factors, genetic resources, antiviral breeding

## Abstract

The ability of plant viruses to propagate their genomes in host cells depends on many host factors. In the absence of an agrochemical that specifically targets plant viral infection cycles, one of the most effective methods for controlling viral diseases in plants is taking advantage of the host plant’s resistance machinery. Recessive resistance is conferred by a recessive gene mutation that encodes a host factor critical for viral infection. It is a branch of the resistance machinery and, as an inherited characteristic, is very durable. Moreover, recessive resistance may be acquired by a deficiency in a negative regulator of plant defense responses, possibly due to the autoactivation of defense signaling. Eukaryotic translation initiation factor (eIF) 4E and eIF4G and their isoforms are the most widely exploited recessive resistance genes in several crop species, and they are effective against a subset of viral species. However, the establishment of efficient, recessive resistance-type antiviral control strategies against a wider range of plant viral diseases requires genetic resources other than eIF4Es. In this review, we focus on recent advances related to antiviral recessive resistance genes evaluated in model plants and several crop species. We also address the roles of next-generation sequencing and genome editing technologies in improving plant genetic resources for recessive resistance-based antiviral breeding in various crop species.

## Introduction

Plant viruses are obligate parasitic microbes that can be characterized by their distinct life cycles depending on host plant machinery. Their genomes are the simplest among plant-associated microbes: a single, or multiple, DNA or RNA molecule(s) encoding several proteins, some of which encapsidate the DNA or RNA to form viral particles. Plant viruses do not deploy specific structures to enter into plant cells and, in general, passively enter through wounds or are transmitted by other organisms including insects, mites, and fungi. Frequent mutations due to error-prone genome replications enable viruses to circumvent plant defense systems and cause severe crop production losses (Kobayashi et al., 2014). Thus far, agrochemicals that directly target viral life cycles have not been developed, and, consequently, it remains difficult to control plant viral diseases. Furthermore, due to worldwide climate change and international trade, there is an increasing risk of plant virus outbreaks.

Great efforts have been made to control plant viral diseases to enhance crop production (Nicaise, 2014; Tsuda and Sano, 2014). Measures used to control these diseases can be categorized into those that depend on plant defense machinery and those that do not. Resistant cultivars, whose traits have been introduced by crossing, are commonly used as crop species to control plant viruses. Plant host resistance is achieved in two ways: one method involves dominant *Resistance* (*R*) genes and the other depends on recessive alleles of genes that are critical for plant viral infection. Most of the dominant *R* genes encode proteins with nucleotide-binding sites and leucine-rich repeats (NB-LRR), and other proteins from the same family confer resistance to bacterial and fungal pathogens (Moffett, 2009; Padmanabhan and Dinesh-Kumar, 2014). In addition, several genes that are distinct from the conventional NB-LRR–type *R* genes have been described (Chisholm et al., 2000; Ishibashi et al., 2007; Yamaji et al., 2012). The second mechanism of plant resistance to viruses, referred to as recessive resistance, is also widely exploited in many crops (Truniger and Aranda, 2009; Wang and Krishnaswamy, 2012). In fact, about half of the alleles responsible for virus-resistance in crops are recessive (Kang et al., 2005). Recessive resistance traits can be introduced into crop species by crossing, or random mutagenesis and selection (Piron et al., 2010). Recessive resistance breeding has the practical advantages of not requiring the introduction of transgenes and not being restricted by the selection of naturally occurring traits only. However, most of the recessive resistance genes isolated to date are eukaryotic translation initiation factors (eIF) 4E and eIF4G, and their isoforms (hereafter eIF4Es).

Mutations in eIF4Es confer loss-of-susceptibility to potyviruses and several other viruses. To enable recessive resistance-based crop breeding against a wide range of plant viruses, it is important to improve the genetic resources available for recessive resistance other than eIF4Es. In the absent of naturally occurring recessive resistant cultivars, and if eIF4Es-mediated resistance is not effective in a plant–virus interaction, a mutation in a potential recessive resistance gene can be introduced. This review focuses on our current understanding of the genetic resources for recessive resistance, and how to enhance them using technologies such as next-generation sequencing (NGS) and genome editing for recessive resistance-based antiviral breeding in various crop species.

## eIf4Es-Mediated Recessive Resistance

Recessive resistance is based on the molecular interactions between viruses and host plants. Plant viruses propagate their genomes in plant cells by hijacking large numbers of host cell proteins, and then spread to adjacent healthy cells and tissues (Hyodo and Okuno, 2014; Wang, 2015). Mutations in the plant genes encoding factors necessary for viral infection can interfere with viral propagation in plants. Another possible mechanism of recessive resistance against plant viruses is based on the autoactivation of plant defense responses when there is a deficiency in a negative regulator of defense signaling (Truniger and Aranda, 2009). However, no experimental evidence has been obtained to directly support the latter hypothesis in naturally occurring cultivars (Orjuela et al., 2013).

Recessive resistance mediated by eIF4Es was first found in mutants of *Arabidopsis thaliana* exhibiting loss-of-susceptibility to tobacco etch virus (TEV; *Potyvirus*), which is due to deficiency in the *eIFiso4E* gene, an isoform of *eIF4E* (Lellis et al., 2002). Subsequent studies revealed that eIF4Es-mediated resistance against potyviruses is found in several resistant crop cultivars including pepper (*Capsicum annuum*), lettuce (*Lactuca sativa*), and wild tomato (*Solanum habrochaites*) (Ruffel et al., 2002, 2005; Nicaise et al., 2003). In addition to potyviruses, eIF4Es-mediated resistance to other viruses has been observed. These include cucumber mosaic virus (CMV; *Cucumovirus*) in *Arabidopsis* (Yoshii et al., 2004); two carmoviruses, turnip crinkle virus (TCV) in *Arabidopsis* (Yoshii et al., 1998) and melon necrotic spot virus (MNSV) in melon (*Cucumis melo*) (Nieto et al., 2006); two bymoviruses, barley mild mosaic virus (BaMMV) and barley yellow mosaic virus (BYMV) in barley (*Hordeum vulgare*) (Kanyuka et al., 2005; Stein et al., 2005); and rice yellow mottle virus (RYMV; *Sobemovirus*) in rice (*Oryza sativa*) (Albar et al., 2006) (this information is also summarized in Truniger and Aranda, 2009 and Sanfaçon, 2015). Unsurprisingly, eIF4Es-mediated resistance is only effective against viruses that interact specifically with at least one of the eIF4Es. Remarkably, in *Arabidopsis*, selective involvement of eIF4Es is found even in closely related viruses in the same genera, including *Potyvirus* and *Polerovirus* (Sato et al., 2005; Nicaise et al., 2007; Reinbold et al., 2013), suggesting that the specific interactions between these viruses and eIF4Es developed after these species diverged from one another. Conservation of translation initiation factors in plants indicates that a wide range of plant viruses may take advantage of host eIF4Es; however, due to partial functional redundancy among eIF4E isoforms, deficiency of an individual in eIF4Es does not always confer resistance to all plant viruses (Mayberry et al., 2011; Martínez-Silva et al., 2012). Moreover, because of the essential roles of eIF4Es in plant viability, knockout mutations of either eIF4E or eIF4G and its corresponding isoform result in an embryo-lethal phenotype (Nicaise et al., 2007; Patrick et al., 2014). Because the utility of eIF4Es as recessive resistance genes is limited, it is important to identify and characterize additional genetic targets that may mediate recessive resistance against a wider range of viral species.

## Positive Regulators of Viral Infection: Genetic Resources for Recessive Resistance

Over the past few decades, a large number of host factors have been isolated and functionally characterized to generate a better understanding of virus life cycles (Nagy and Pogany, 2011; Hyodo and Okuno, 2014; Wang, 2015). To identify host factors, forward and reverse genetic approaches using *Arabidopsis* and other model plants have been used (Ishikawa et al., 1991; Yoshii et al., 2009; Castelló et al., 2010). In addition, other host factors have been identified by screening for interactors with viral proteins and components of protein complexes containing viral factors (Mine et al., 2010; Nishikiori et al., 2011; Xiong and Wang, 2013). Genome-wide screening using the heterologous yeast system with brome mosaic virus (BMV; *Bromovirus*) and also with tomato bushy stunt virus (TBSV; *Tombusvirus*), has revealed that viral infections are affected by more than 100 host genes in each case; these genes encode a distinct set of host factors for each of the two viruses (Kushner et al., 2003; Gancarz et al., 2011; Nagy, 2016). Among the identified host proteins, several give rise to loss-of-susceptibility phenotypes when the corresponding genes are mutated. In addition, other host proteins identified from naturally occurring resistant cultivars are important genetic resources for recessive resistance. They are discussed separately in the next section.

With several exceptions (Fujisaki and Ishikawa, 2008; Cheng et al., 2009; Huh et al., 2013), many of the host factors characterized so far in plants positively control viral infection; herein, we refer to them as “positive regulators.” These positive regulators have been characterized predominantly through transient knockdown experiments. Knockdown of a gene encoding a positive regulator of viral infection results in a decrease of viral accumulation. This phenotype is equivalent to recessive resistance, and leads us to expect that the corresponding host factor could be a recessive resistance gene in crop species, especially if deficiency of the host factor has no adverse effect on plant growth. However, there could be a qualitative difference between the transient knockdown of a host factor by RNA silencing and the null mutation. When a host factor is indispensable for plant viability or is encoded by functionally redundant genes, the transient knockdown of the factor and the null mutation may produce different phenotypes (Wei et al., 2013; Xiong and Wang, 2013). Alternatively, even if a host factor plays an essential role in plant viability, a conserved amino acid substitution could confer viral resistance without an adverse effect on plant growth (Ouibrahim et al., 2014). This scenario would suggest that the substituted amino acid is critical for molecular plant–virus interaction, but not for plant viability. Further molecular analyses will be necessary to reveal the availability of positive regulators as recessive resistance genes.

Some of the positive regulators identified so far are common among distantly related viruses (Nagy et al., 2014). Although confirmatory molecular studies will be required, deficiency of these host factors could generate recessive resistance against a wide range of viruses. For example, HSP90 is required for viral replication of red clover necrotic mosaic virus (RCNMV; *Dianthovirus*) (Mine et al., 2012) and bamboo mosaic virus (BaMV; *Potexvirus*) (Huang et al., 2012). Infection by rice stripe virus (RSV; *Tenuivirus*) (Jiang et al., 2014), turnip mosaic virus (TuMV; *Potyvirus*) (Jungkunz et al., 2011) and RCNMV (Mine et al., 2012) is not supported efficiently after silencing of HSP70. eEF1A seems to be commonly involved in viral replication via interaction with a viral replicase in tobacco mosaic virus (TMV; *Tobamovirus*) (Yamaji et al., 2006, 2010), TuMV (Thivierge et al., 2008), and TBSV (Li et al., 2009) as well as with viral RNA in turnip yellow mosaic virus (TYMV; *Tymovirus*) (Dreher et al., 1999), TMV (Zeenko et al., 2002), and TBSV (Li et al., 2009). Noted that these host factors are also involved in plant growth, gene expression, and plant hormone signaling (Ransom-Hodgkins, 2009; Clément et al., 2011; Jungkunz et al., 2011; Zhang X.C. et al., 2015). In plants, cytosolic HSP70 and HSP90 are important for disease resistance against pathogens other than viruses (Kanzaki et al., 2003; Takahashi et al., 2003). Therefore, some mutations of these genes not only confer recessive resistance to a plant virus but may also have unexpected adverse effects on plants.

## Promising Genetic Resources for Recessive Resistance

If a host factor for viral infection can be mutated in one plant species without any adverse effects on plant growth at least under controlled greenhouse conditions, one would expect that this might be possible in other plant species, too, and that such host factors would be promising genetic targets for recessive resistance. In this section, we discuss host factors that have been identified as potential targets for recessive resistance either from loss-of-susceptibility mutants or from naturally occurring resistant cultivars (**Table 1**). It is noteworthy that some translation factors, including polyA-binding protein (PABP) and DEAD-box RNA helicase (referred to as DDXs or RHs), are promising genetic targets for recessive resistance (Dufresne et al., 2008; Li et al., 2016), but because they have been discussed in detail elsewhere (Sanfaçon, 2015), they are not covered in this section.

**Table 1:** The genetic resources for recessive resistance found in loss-of-susceptibility mutants and naturally occurring resistant cultivars.

Gene	Plant species encoding homologs	Cause of resistance	Affected virus^1^	Non-affected virus^1^	Reference
*TOM1*; *TOM3*	*Nicotiana* spp. *Solanum lycopersicum Capsicum annuum Oryza sativa*	Loss-of-susceptibility by ethyl methanesulfonate (EMS) mutagenesis	YoMV ToMV TMV TMGMV PMMoV	CMV TCV TYMV	Ishikawa et al., 1991 Ishikawa et al., 1993 Yamanaka et al., 2000 Yamanaka et al., 2002 Kumar et al., 2012
*TOM2A*; *TOM2B*	*Arabidopsis thaliana*	Loss-of-susceptibility by fast neutron mutagenesis	YoMV ToMV	CMV TCV TYMV	Ohshima et al., 1998 Tsujimoto et al., 2003
*ARL8*	*Arabidopsis thaliana Nicotiana tabacum*	Loss-of-susceptibility by simultaneous null mutation of *ARL8a* and *ARL8b* by T-DNA insertions	ToMV YoMV	CMV	Nishikiori et al., 2011
*RIM1*	*Oryza sativa Arabidopsis thaliana*	Loss-of-susceptibility by Tos17-based insertional mutagenesis	RDV	RTYV RSV	Yoshii et al., 2009
*DBP1*	*Arabidopsis thaliana Nicotiana tabacum Zea mays Oryza sativa Mesembryanthemum crystallinum*	Loss-of-susceptibility in a T-DNA mutant	TuMV PPV	CMV	Carrasco et al., 2005 Castelló et al., 2010
*cPGK*	*Nicotiana* spp. *Solanum lycopersicum Solanum tuberosum Populus trichocarpa Sorghum bicolor Oryza sativa Triticum aestivum Zea mays*	Natural resistance gene, *rwm1*, found in *Arabidopsis* Cvi-0 ecotype	WMV PPV BaMV	PVX CMV	Lin et al., 2007 Ouibrahim et al., 2014 Poque et al., 2015
*EXA1*	*Arabidopsis thaliana Oryza sativa Solanum lycopersicum*	Loss-of-susceptibility by EMS mutagenesis	PlAMV PVX AltMV	CMV TCV YoMV	Hashimoto et al., 2016
*PVIP1; PVIP2*	*Arabidopsis thaliana Pisum sativum Nicotiana benthamiana*	Loss-of-susceptibility in a knockdown mutant of each *PVIP*	TuMV	-	Dunoyer et al., 2004
*PDLP1; PDLP2; PDLP3*	*Arabidopsis thaliana*	Loss-of-susceptibility by triple mutation of *PDLP1, PDLP2* and *PDLP3* by T-DNA insertions	GFPV CaMV	ORMV	Amari et al., 2010
*PCaP1*	*Arabidopsis thaliana*	Loss-of-susceptibility in a T-DNA mutant	TuMV	ORMV	Vijayapalani et al., 2012
*SYTA*	*Arabidopsis thaliana*	Loss-of-susceptibility in a T-DNA mutant	CaLCuV TVCV TuMV	CaMV	Lewis and Lazarowitz, 2010 Uchiyama et al., 2014
*Sec24a*	*Arabidopsis thaliana*	Loss-of-susceptibility in an EMS-induced mutant	TuMV	-	Jiang et al., 2015
*RHD3*	*Arabidopsis thaliana*	Loss-of-susceptibility in a T-DNA mutant	TSWV	-	Feng et al., 2016
*PDIL5-1*	All plant species	Natural resistance gene, *rym11*, found in barley	BaYMV BaMMV	-	Yang et al., 2014
*IRE1*	All plant species	Loss-of-susceptibility by double mutation of *IRE1a* and *IRE1b* by T-DNA insertions	TuMV	-	Zhang L. et al., 2015
*bZIP60*	All plant species	Loss-of-susceptibility in a T-DNA mutant	TuMV PVX	-	Ye et al., 2011 Zhang L. et al., 2015
*HAT1; HAT2; HAT3*	*Arabidopsis thaliana*	Loss-of-susceptibility by triple mutation of *HAT* genes by T-DNA insertions	CMV	-	Zou et al., 2016
*CPR5*	*Oryza glaberrima Arabidopsis thaliana*	Natural resistance gene, *rymv2*, found in African rice	RYMV	-	Orjuela et al., 2013

*^1^Virus abbreviations not provided in the text: TMGMV (tobacco mild green mosaic virus; *Tobamovirus*), PMMoV (pepper mottle mosaic virus, *Tobamovirus*), AltMV (alternanthera mosaic virus; *Potexvirus*).*

Tobamovirus multiplication 1 (TOM1) has been identified using *Arabidopsis* mutants with loss-of-susceptibility to youcai mosaic virus [YoMV; *Tobamovirus* (previously referred to as TMV-Cg)] (Yamanaka et al., 2000). The *tom1-1* mutation does not completely suppress YoMV accumulation unless the *TOM3* gene is also mutated (Yamanaka et al., 2002). However, CMV and TCV accumulation are unaffected in the *tom1tom3* double mutant (Yamanaka et al., 2002). TOM1 and TOM3 are closely related, seven-pass membrane proteins, and TOM1 interacts with the helicase domain of YoMV replicase (the current model of tobamovirus replication is well documented in another review; Ishibashi and Ishikawa, 2016). Although *TOM1* and *TOM3* homologs are encoded in *Nicotiana* spp., tomato (*S. lycopersicum*), pepper and rice (Kumar et al., 2012), functional validation of these proteins in tobamovirus accumulation has only been performed in *Nicotiana* spp. (Asano et al., 2005; Chen et al., 2007). Asano et al. (2005) demonstrated that knockdown of both *TOM1* and *TOM3* genes in *N. tabacum* completely suppresses three distinct tobamoviruses other than YoMV. The genes identified from the *tom2-1 Arabidopsis* mutant are *TOM2A* and *TOM2B* (Tsujimoto et al., 2003). TOM2A is a four-pass membrane protein and TOM2B is a basic protein. Although the molecular function of TOM2B is unknown, TOM2A is thought to be involved in tobamovirus accumulation via its interaction with TOM1 (Tsujimoto et al., 2003; Ishibashi and Ishikawa, 2016).

ARL8, a small GTP-binding ARF-family protein, has been co-purified with a replicase from tomato mosaic virus (ToMV; *Tobamovirus*) (Nishikiori et al., 2011). ARL8, together with TOM1, is involved in ToMV replication through regulating the enzymatic activity of a ToMV replicase in RNA synthesis and capping (Nishikiori et al., 2011). While a deletion in any one of three *ARL8* genes does not alter ToMV accumulation in *Arabidopsis*, mutation of both the *ARL8a* and *ARL8b* genes completely suppressed viral accumulation without any adverse effect on plant growth (Nishikiori et al., 2011). *ARL8* demonstrates that host factor genes and their functionally redundant homologs may be good targets for joint mutations that together produce recessive resistance. Alternatively, as demonstrated by the eIF4Es (Sato et al., 2005; Nicaise et al., 2007; Reinbold et al., 2013), when a distinct protein among a functionally related group has established a specific interaction with a virus, the corresponding gene alone could be targeted for mutation to generate recessive resistance.

*Rice dwarf virus multiplication 1 (rim1)* mutant is produced by a retrotransposon Tos17 insertion in an NAC-domain transcription factor and shows loss-of-susceptibility to rice dwarf virus (RDV; *Phytoreovirus*) (Yoshii et al., 2009). However, *rim1* mutants are susceptible to two other rice viruses, rice transitory yellowing virus (RTYV; *Rhabdovirus*) and RSV (Yoshii et al., 2009). The RIM1 protein is closely related to an *Arabidopsis* NAC domain protein, ANAC028. Yoshii et al. (2009) also demonstrated that the *rim1* mutation has a small negative effect on the survival of green rice leafhopper (*Nephotettix cincticeps*), an insect vector of RDV. This may be related to observations of jasmonic acid (JA)-induced phenotypes in some *rim1* mutants (Yoshii et al., 2010). Although the molecular function of RIM1 in RDV infection is unclear, the protein could be critical for RDV infection without being a general defense repressor if RIM1-mediated resistance is specific for RDV (Yoshii et al., 2009).

Knockout mutation in *DNA-binding protein phosphatase 1* (*DBP1*) gene does not influence plant growth in *Arabidopsis*, but does result in resistance to two potyviruses, TuMV and plum pox virus (PPV) (Castelló et al., 2010). The domain structure of DBP1 suggests that it functions in signal transduction as well as in transcriptional regulation (Carrasco et al., 2006). *DBP1*-related genes are present in dicotyledons and monocotyledons, including *N. tabacum*, maize (*Zea mays*), and rice (Carrasco et al., 2005). As DBP1 forms a stabilizing interaction with eIFiso4E, the loss of susceptibility of *dbp1* mutants may be related to the low-level accumulation of eIFiso4E (Castelló et al., 2010). DBP1 also interacts with 14-3-3 family protein GRF6, regulating its phosphorylation status, and the *grf6* mutant is resistant to PPV (Carrasco et al., 2006, 2014). The DBP1 interaction with GRF6 may regulate the phosphorylation status of eIFiso4E, thereby altering its cap-binding activity (Khan and Goss, 2004). Further studies are needed to confirm the mechanism of DBP1-mediated resistance.

A recessive allele conferring resistance to watermelon mosaic virus (WMV; *Potyvirus*) has been identified in the *Arabidopsis* ecotype Cvi-0 and designated *resistance to watermelon mosaic virus 1* (*rwm1*) (Ouibrahim et al., 2014). Map-based cloning identified an amino acid substitution in a nuclear-encoded chloroplast phosphoglycerate kinase, cPGK2 (Ouibrahim et al., 2014). *cPGK2* gene homologs are found in dicotyledons and monocotyledons including: *Nicotiana* spp., tomato, potato (*S. tuberosum*), poplar (*Populus trichocarpa*), sorghum (*Sorghum bicolor*), rice, wheat (*Triticum aestivum*), and maize. Downregulation of *cPGK* genes in *N. benthamiana* compromises WMV (Ouibrahim et al., 2014) and PPV accumulation (Poque et al., 2015). Remarkably, cPGK is associated with the 3′-untranslated region of BaMV genomic RNA and is required for the efficient accumulation of BaMV in *N. benthamiana* (Lin et al., 2007). Recently, Cheng et al. (2013) demonstrated that cPGK recruits BaMV genomic RNA to chloroplasts to support BaMV replication in *N. benthamiana*. Consistent with this, some potyviruses are thought to replicate their genomic RNA in chloroplasts (Wei et al., 2013). Further studies are needed to reveal the role of cPGK in potyvirus and potexvirus infection.

More recently, Hashimoto et al. (2016) demonstrated that deficiencies in *essential for potexvirus accumulation 1* (*EXA1*) gene were present in a loss-of-susceptibility *Arabidopsis* mutant that did not support plantago asiatica mosaic virus (PlAMV; *Potexvirus*) accumulation. *EXA1* is an unannotated gene in plants, but contains a putative eIF4E-binding motif and a GYF domain, which binds to proline-rich peptides (Kofler and Freund, 2006). Based on sequence comparisons with other related genes, *EXA1* homologs are encoded in rice and tomato and are structurally related to human GIGYF2 protein (Hashimoto et al., 2016). T-DNA insertion of *EXA1* gene, forming *exa1-1* mutant, does not affect accumulation of CMV, TCV, or YoMV, but does suppress the accumulation of two distinct potexviruses other than PlAMV (Hashimoto et al., 2016). Because human GIGYF2 regulates mRNA translation (Morita et al., 2012), it is conceivable that EXA1 might also regulate the translation of a viral protein during early infection. Further studies are needed to reveal the role of EXA1 in virus infection and whether EXA1-mediated resistance is effective in other plant species and against viruses other than potexviruses.

Functional studies on the host factors that play a critical role in viral transport to healthy plant cells have shed light on several potential recessive resistance genes conferring resistance to plant viruses. Once the viral genomes are replicated in the initially infected cells, the viruses must transport their genomes through plasmodesmata (PD), which are plant-specific intercellular nanopores that connect neighboring cells. To transport infectious entities to PD, viral movement proteins (MPs) recruit host factors and host machineries, such as cellular trafficking pathways. Viruses that are able to reach the phloem by continuous transport to neighboring cells systemically spread through the sieve tube, depending on host factors. Potyvirus VPg-interacting protein from pea (PVIPp) was isolated through yeast two-hybrid screening of a cDNA library from pea (*Pisum sativum*). PVIPp interacts with VPg protein of pea seed-borne mosaic virus (PSbMV; *Potyvirus*) (Dunoyer et al., 2004). In *Arabidopsis*, PVIP1 and PVIP2 are closely related homologs, and their knockdown in plants confers loss-of-susceptibility to TuMV (Dunoyer et al., 2004). A TuMV mutant with a point mutation in VPg that affects the interactions with PVIP1 compromises cell-to-cell transport (Dunoyer et al., 2004). Since PVIP1 and PVIP2 interact with VPg proteins of other potyviruses, PVIPs-mediated resistance may also be effective against other potyviruses. *Arabidopsis* PCaP1 and COPII coatomer Sec24a interact with P3N-PIPO and 6K_2_ of TuMV, respectively (Vijayapalani et al., 2012; Jiang et al., 2015). Both host factors are involved in distinct steps in TuMV cell-to-cell transport. A mutation in *PCaP1* or *Sec24a* gene in *Arabidopsis* impairs TuMV infection (Vijayapalani et al., 2012; Jiang et al., 2015). Knockout of *root hair defective 3* (*RHD3*), whose gene product is involved in the formation of the tubular ER network structure, significantly inhibits the systemic infection of tomato spotted wilt virus (TSWV; *Tospovirus*) (Feng et al., 2016). PD-located protein 1 (PDLP1) was originally identified as a cell wall-associated membrane protein in *Arabidopsis* and was isolated from a highly purified cell wall fraction (Bayer et al., 2006; Thomas et al., 2008). A *PDLP1, PDLP2* and *PDLP3* triple mutant inhibits systemic infection of grapevine fanleaf virus (GFLV; *Nepovirus*) and cauliflower mosaic virus (CaMV; *Caulimovirus*) but not oilseed rape mosaic virus (ORMV; *Tobamovirus*) (Amari et al., 2010). Although GFLV and CaMV are distantly related viruses, MPs of both viruses form a specific structure, called a tubule used in cell-to-cell transport. These results imply that the loss-of-susceptibility of *pdlp1/2/3* triple mutant is also applicable to other viruses that employ the tubule-based transport strategy. *Arabidopsis* synaptotagmin (SYTA), a plant homolog of calcium sensors widely studied in animals, has been shown to interact with MP of cabbage leaf curl virus (CaLCuV; *Begomovirus*) (Lewis and Lazarowitz, 2010). Remarkably, a *syta* mutant significantly inhibits systemic infection of a diverse spectrum of plant viruses, including CaLCuV, turnip vein clearing virus (TVCV; *Tobamovirus*) and TuMV, but not of CaMV (Lewis and Lazarowitz, 2010; Uchiyama et al., 2014), suggesting that SYTA and the involved cellular machinery are promising candidates for recessive resistance against a wide range of viruses. In spite of the above-mentioned results, no study has reported a naturally occurring recessive resistant cultivar that targets viral transport. Thus, the targeting of viral transport for recessive resistance may well be technically challenging.

The unfolded protein response (UPR) is a highly conserved cellular machinery that allows both animals and plants to cope with an overload of unfolded proteins in the endoplasmic reticulum (ER) (Howell, 2013). Recently, several studies have suggested the relevance of the UPR in plant–virus interactions (Ye et al., 2011; Yang et al., 2014; Zhang L. et al., 2015; Arias Gaguancela et al., 2016). In barley, the recessive resistance genes *rym4/rym5*, which are alleles of *eIF4E*, have been overcome by resistance-breaking isolates of BaMMV and BaYMV (Hariri et al., 2003; Kühne et al., 2003), whereas *rym11* resistance cultivars are highly durable against both virus isolates. Positional cloning has revealed that a mutation in *protein disulfide isomerase like 5-1* (*PDIL5-1*) is responsible for the recessive resistance gene *rym11* (Yang et al., 2014). The natural variation among *HvPDIL5-1* genes suggests that most of the *rym11* cultivars collected from eastern Asia are the result of frequent interactions with highly divergent forms of BaMMV and BaYMV (Yang et al., 2014). PDIL5-1 is a conserved protein in plants and animals, which functions as an endoplasmic reticulum-localized chaperone in the UPR (Howell, 2013). *Arabidopsis bzip60-2* mutant and *ire1a/ire1b* double mutant, which are mutants of other UPR components, show loss-of-susceptibility to TuMV (Zhang L. et al., 2015). Silencing of *bZIP60* gene significantly suppresses the accumulation of potato virus X (PVX; *Potexvirus*) in *N. benthamiana* (Ye et al., 2011). Although the mechanism of the resistance mediated by the UPR components remains unclear, the striking conservation of UPR components and the consistency of their roles in viral infection imply that they are promising genetic targets for recessive resistance to a wide range of viruses.

Several lines of evidence suggest that a mutation in a gene encoding a component of plant defense responses could confer resistance to viruses. *Arabidopsis ssi2* mutant, which accumulates high levels of plant defense hormone salicylic acid (SA), confers resistance to CMV (Sekine et al., 2004). Based on the experimental evidences, Sekine et al. (2004) demonstrated that the resistance to CMV in *ssi2* mutant is unrelated to SA production and the dwarf phenotype. Some *Arabidopsis* mutants related to the defense hormone ethylene, such as *acs6* mutant, also shows resistance to YoMV (Chen et al., 2013). Although the loss-of-susceptibility of the mutants related to defense responses may be due to elevated antiviral defense signaling(s), mutants such as *ssi2* mutant frequently show an abnormal growth phenotype (Sekine et al., 2004). Remarkably, a triple mutant of *homeodomain-leucine zipper protein 1* (*HAT1*) and its related genes *HAT2* and *HAT3* confers loss-of-susceptibility to CMV without any growth defect despite the high level of SA and JA accumulation (Zou et al., 2016). However, as discussed earlier, if a deficiency in a specific defense signaling molecule confers recessive resistance to a plant virus, there could be unexpected adverse effects on the plants because of the complex nature of the plant defense signaling network (Mine et al., 2014). The *RYMV2* gene, identified using the resistant Tog7291 accession of African rice (*O. glaberrima*), encodes a recessive resistance gene that is responsible for durable resistance to rice yellow mottle virus (RYMV; *Sobemovirus*). The *rymv2* mutant is deficient in a rice homolog of the *Arabidopsis CPR5* gene (Orjuela et al., 2013), which has a repressive role in plant defense responses (Yoshida et al., 2002). Alleles of the *rymv2* mutant have also been found in seven additional African rice accessions that were resistant to RYMV (Orjuela et al., 2013). Due to the role of *Arabidopsis* CPR5 in defense responses, the activation of defense responses by *rymv2* alleles presumably contributes to RYMV resistance.

## Strategies for Improving the Genetic Resources for Recessive Resistance

Despite their importance, few host factors have successfully been identified by forward genetic screening or as naturally occurring recessive resistant alleles (**Table 1**). In part, this is because genetic screening and traditional gene mapping approaches are labor intensive and costly; it is also difficult to identify particular types of gene (for example, those that are functionally redundant or those that are essential for plant viability) using a genetic approach. Moreover, even after genes of interest have been identified, there may be substantial delays before these can be used to generate recessive resistance in crop species. Establishing resistant cultivars targeting a specific gene using random mutagenesis and screening, and introducing traits through crossing, are both technically challenging and time-consuming procedures. However, the emergence of NGS, genome editing, and other technologies have provided new opportunities for improving and utilizing genetic resources for recessive resistance breeding.

As discussed above, loss-of-susceptibility to viral infection produced by random mutagenesis is genetically equivalent to recessive resistance found in natural variants. Performing random mutagenesis in crops and model plants circumvents the limitations imposed by relying on genetic variation found only in naturally occurring cultivars. In addition, the recessive resistance discussed earlier including that mediated by eIF4Es, is effective in several plant species. Therefore, random mutagenesis and selection for loss-of-susceptibility mutants in model plants, including *Arabidopsis*, is still an attractive option for improving genetic resources to apply recessive resistance in crops. Model plants facilitate the isolation of loss-of-susceptibility mutants and the subsequent identification of corresponding genes due to the availability of whole-genome sequence information and their characteristically simple genetics (Yamanaka et al., 2000; Yoshii et al., 2009). By contrast, random mutagenesis performed in polyploid plants (e.g., wheat and soybean) presents difficulties that include obtaining mutants with discernible phenotypes, often due to functional complementation by redundant genes. However, to ultimately apply mutant screening in *Arabidopsis* to recessive virus resistance-based crop breeding, it is important to select a virus species that can infect *Arabidopsis*, and comes from the same viral genus as the target virus (Ishikawa et al., 1991; Fujisaki et al., 2004; Yamaji et al., 2012). Additionally, to reliably and rapidly detect viral infection, the introduction of green fluorescent protein into an infectious viral clone is desirable (Baulcombe et al., 1995; Minato et al., 2014). The rationale for this is based on the expectation that viruses from the same genus have similar life cycles. In fact, it is known that some host factors, including eIF4Es, play a similar role in infection by different viruses from the same genus (Asano et al., 2005; Ouibrahim et al., 2014; Yang et al., 2014; Poque et al., 2015; Hashimoto et al., 2016). However, there are many exceptions that challenge this rationale (for example, see the section on eIF4Es-MEDIATED RECESSIVE RESISTANCE). Thus, validation of the results obtained from *Arabidopsis* mutant screening in other host–virus interactions is essential.

Next-generation sequencing technologies have made it easy for many plant scientists to access whole-genome plant sequencing (Morrell et al., 2012). Simultaneously, genomics-based crop breeding using NGS technologies is expected to overcome the challenge of feeding an increasing world population. As suggested by Varshney et al. (2014), NGS technologies, which have rarely been applied to antiviral breeding using natural variants (Zuriaga et al., 2013; Mariette et al., 2016), would be quite useful for identifying loci in naturally resistant variants and also for breeding to introduce resistant loci into specific cultivars. Several studies have identified loci of interest from *Arabidopsis* mutants using whole-genome sequencing of pooled mutant F2 populations (Schneeberger et al., 2009; Austin et al., 2011; Uchida et al., 2011). The *EXA1* gene was identified successfully from a loss-of-susceptibility mutant by combining conventional map-based cloning and whole-genome sequencing of mutant plants (Hashimoto et al., 2016). Methods based on a similar concept have also been established in rice (Abe et al., 2012; Takagi et al., 2013). These studies suggest that a resistance locus could be identified rapidly from a loss-of-susceptibility mutant based on whole-genome sequencing in *Arabidopsis* and rice.

Genome editing based on sequence-specific nucleases such as zinc-finger nucleases (ZFNs), transcription activator-like effectors (TALENs), and CRISPR-associated protein 9 (Cas9) in clustered, regularly interspaced, short palindromic repeat (CRISPR)/Cas systems has recently been developed to enable targeted mutagenesis and gene insertion in eukaryotic genomes (Gaj et al., 2013). The applications of these genome editing technologies in plants are well summarized elsewhere (Araki and Ishii, 2015; Luo et al., 2016; Ma et al., 2016). Importantly, genome editing technologies have been employed not only in model plants but also in several crop species, and they are now being applied even more widely. One of the outstanding points of genome editing in terms of its application to crop breeding is that the original transgenes for genome editing can be removed via segregation after editing. Recently, the CRISPR/Cas9 system was used to establish eIFiso4E-deficient *Arabidopsis* mutants that were free from transgenes and exhibited recessive resistance to TuMV (Pyott et al., 2016). More importantly, the CRISPR/Cas9 system was also applied to cucumber (*Cucumis sativus*) to disrupt the *eIF4E* gene, and the non-transgenic, eIF4E-deficient plant lines were resistant to the cucumber vein yellowing virus (CVYV; *Ipomovirus*) and two potyviruses (Chandrasekaran et al., 2016). Even in polyploid soybean, duplicated genes have been mutagenized using ZFNs (Curtin et al., 2011). In allohexaploid wheat, simultaneous mutation of three *MILDEW RESISTANCE LOCUS* genes by TALENs resulted in resistance to a powdery mildew fungal pathogen (Wang et al., 2014). Editing of multiple genes using the CRISPR-Cas9 system is applicable to *Arabidopsis* and rice (Ma et al., 2015). These studies suggest that genetic targets for recessive resistance may be mutagenized in various crop species (including polyploid crops) using genome editing technologies. Previously, the only methods for introducing a recessive resistant locus from a natural variant into a specific cultivar were crossing and random mutagenesis. Because targeted mutagenesis by genome editing involves a small deletion or insertion in a specific genomic site through non-homologous end-joining (NHEJ), genome editing technologies are compatible with developing and applying genetic resources for recessive resistance in crop species.

## Conclusion and Future Perspectives

In this article, we focused on emphasizing the importance of recessive resistance in future anti-viral breeding. Significant fundamental research efforts have been invested in identifying host factors involved in plant virus infection. The corresponding genes are potential targets for recessive resistance, in addition to the eIF4Es. The application of this information to crop research should result in the development of new recessive resistancetraits. However, to avoid the unforeseeable effects of mutations and to expand the possible application range of each host factor, further studies are essential and should focus on the molecular function of each factor in viral infection and also in that of the relevant viruses. In addition, it is necessary to identify plants that are susceptible to the viruses through each particular host factor. Currently, extensive characterization studies have been limited to only a few plant virus species, and so it is critical to expand this research to include other viruses with agricultural impact (Rybicki, 2015). Genome editing technologies are promising methods for introducing recessive resistance into various crop species. Moreover, some genome-edited crops have already been made available without restriction by the US Department of Agriculture, one of the agencies responsible for the regulation of genetically modified organisms (GMOs) in the USA (Waltz, 2016a,b). However, it remains unclear whether resources created by genome editing are subject to regulations associated with GMOs in other countries (Hartung and Schiemann, 2014; Araki and Ishii, 2015). Based on the possible regulatory guidelines that take into account mutation patterns and modification mechanisms, as suggested by Araki and Ishii (2015), mutation mechanisms capable of producing recessive resistance should be prioritized into categories that may be most easily accepted. Further research to support and enhance the safety of genome editing technologies for recessive resistance-based crop breeding is extremely important.

## Author Contributions

MH, YY, and SN designed the research. MH, YN, and YY surveyed and discussed on the previous researches. MH, YY, and SN wrote the paper with the support by YN.

## Conflict of Interest Statement

The authors declare that the research was conducted in the absence of any commercial or financial relationships that could be construed as a potential conflict of interest.
